# The link between inflammatory/ SCFA profiles and oral/gut microbiome: an observational study in patients with ST-segment elevation myocardial infarction

**DOI:** 10.1016/j.crmicr.2025.100423

**Published:** 2025-06-15

**Authors:** Luis A. Constantino-Jonapa, Oscar R. Aguilar-Villegas, Paulina Hernández-Ruiz, Alma R. Escalona-Montaño, Marco Pallecchi, Héctor González-Pacheco, Gianluca Bartolucci, Simone Baldi, Luis M. Amezcua-Guerra, Amedeo Amedei, M. Magdalena Aguirre-García

**Affiliations:** a Unidad de Investigación UNAM-INC, División de Investigación, Facultad de Medicina, UNAM, Instituto Nacional de Cardiología Ignacio Chávez, Mexico City 14080, Mexico; b Department of Neuroscience, Psychology, Drug Research and Child Health NEUROFARBA, University of Florence, Florence, Italy; c Coronary Care Unit, Instituto Nacional de Cardiología Ignacio Chávez, Mexico City 14080, Mexico; d Department of Immunology, Instituto Nacional de Cardiología Ignacio Chávez, Mexico City 14080, Mexico; e Department of Experimental and Clinical Medicine, University of Florence, 50134 Florence, Italy; f Network of Immunity in Infection, Malignancy and Autoimmunity (NIIMA), Universal Scientific Education and Research Network (USERN), 50139 Florence, Italy

**Keywords:** STEMI, Oral microbiome, Gut Microbiome, SCFAs, Cytokines

## Abstract

Acute myocardial infarction (AMI) is a leading cause of mortality in Mexico. The microbiota plays a crucial role in immune regulation, and its dysbiosis promotes low-grade inflammation, a key contributor to AMI development. This study aimed to compare the oral (OM) and gut microbiota (GM) composition in patients with ST-segment elevation myocardial infarction (STEMI) and healthy controls. Additionally, we explored the interaction between these microbiomes and their correlations with inflammatory profiles and metabolites. In this study, we included 36 STEMI patients and 12 healthy subjects. The composition of both GM and OM was analyzed through 16S sequencing of dental plaque and stool samples. Short-chain fatty acids (SCFAs) were measured via gas chromatography-mass spectrometry, while serum cytokines were assessed using flow cytometry.

STEMI patients exhibited significant differences in OM alpha and beta diversity, while GM structure remained unchanged compared to healthy group. Several differentially abundant genera were identified in both OM and GM. Regarding SCFA profiles, Healthy subjects displayed a higher abundance of isovaleric acid, whereas isobutyric and 2-methylbutyric acids were significantly higher in STEMI patients. Moreover, correlations between circulating SCFAs, cytokines, and microbiota composition were observed in both niches. Furthermore, network analysis suggests that oral bacteria, particularly those linked to periodontal disease, can potentially influence GM by interacting with SCFA-producing bacteria.

This is the first study in México to comprehensively explore OM and GM in STEMI patients. Our findings highlight the potential for developing preventive strategies against myocardial infarction by exploring both oral and gut microbiomes, as well inflammatory markers and SCFAs.

## Highlights

• Oral bacteria like *Lautropia* and *Streptococcus* species were enriched in healthy subjects, while several caries-associated bacteria, like *Veilonella* and *Leptottrichia* and were enriched in STEMI patients.
• *Collinsela* and *Escherichia-Shigella* were associated in the gut microbiome of STEMI patients.
• *Dialister, Gemella, Granulicatella, Haemophilus, [Peptococcaceae] uncultured, Prevotella 2, Rothia, Ruminococcaceae UCG-014, Streptococcus*, and *Veillonella* were found in both oral and gut microbiomes.
• Using Deseq2 analysis, several oral SCFA producing bacteria were associated with STEMI, while in gut microbiome, SCFA-producing bacteria were associated with healthy patients.
• Key bacteria were identified that potentially modulate the microbiome along the oral-gut axis, such as *Veillonella* spp. and *Prevotella*.

## Graphical Abstract

**Figure:**
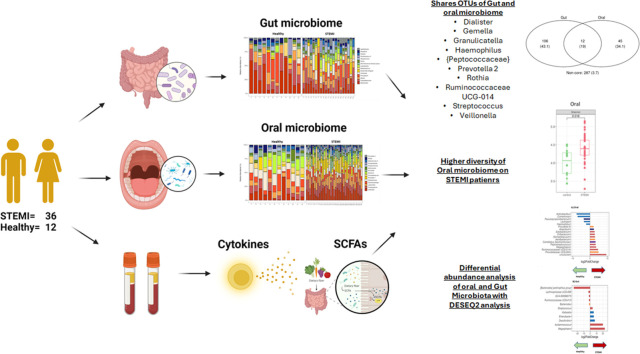

## 1 Introduction

Coronary artery disease (CAD) is a prevalent cardiac condition characterized by the development of atherosclerotic plaques in the lumen of blood vessels, resulting in impaired blood flow and reduced oxygen supply to the myocardium. ST-segment elevation acute myocardial infarction (STEMI) is the most severe form of acute coronary syndrome (ACS) and accounts for a significant portion of mortality worldwide (Gao et al. 2020; Li et al. 2017). The genetic background and environmental factors of the Mexican population predispose to metabolic diseases such as diabetes, hypertension and obesity, which are substantial risk factors for STEMI (Borrayo-Sanchez et al. 2018). Inflammation plays a critical role in STEMI, and in detail, there are four proposed mechanisms: i) plaque rupture with macrophage infiltration and elevated markers of systemic inflammation, ii) plaque fissure without inflammation, iii) plaque erosion without inflammation, and iv) STEMI without vascular thrombosis. In Mexico, 64.8% of urgent cases are diagnosed with STEMI due to plaque rupture, which is characterized by the infiltration of M1 macrophages into the atherosclerotic plaque and the production of inflammatory cytokines such as interleukin (IL)-1β, IL-17, and tumor necrosis factor (TNF).

Multiple factors contribute to the inflammation underlying STEMI, and in recent years, the role of the gut microbiome (GM) in CAD has been extensively studied. (Crea and Libby 2017). A balanced GM is crucial for maintaining host’ health; however, factors such as age, diet and diseases can alter its composition, leading to dysbiosis (Terruzzi and Senesi 2020). GM dysbiosis induces a proinflammatory state in the host by compromising the integrity of the intestinal epithelial barrier, which allows lipopolysaccharides (LPS) and other microbial metabolites to leak into the bloodstream (Piccioni et al. 2021). This imbalance plays a critical role in CAD pathogenesis by inducing a pro-inflammatory state that promotes atherosclerosis. Furthermore, research indicates that short-chain fatty acids (SCFAs), which are primarily produced by the GM through the fermentation of dietary fibers in the intestinal lumen, exhibit cardioprotective properties. In addition to their role in modulating the immune system and maintaining the integrity of the intestinal barrier, SCFAs have been linked to heart health. GM dysbiosis, characterized by a decline in SCFA-producing bacteria, is associated with an increased risk of heart injury. Finally, probiotics containing SCFA-producing bacteria, such as *Akkermansia* spp. and *Bifidobacterium* spp., have been demonstrated to help reduce heart damage (Tang et al. 2023; He et al. 2022).

On the other hand, also the oral microbiome (OM) has been linked to CADs. In the Atherosclerosis Risk in Communities Study (ARIC), patients were followed up to 15 years and OM was identified as risk factor for ischemic stroke. On the other hand, regular dental care was protective, decreasing ischemic stroke. Poor dental care can lead to periodontal diseases, gingivitis and caries. This allows the growth of pathogenic bacteria like *Veillonella* and *Fusobacterium*, generating a local inflammation, allowing oral pathogens and their metabolites to enter the bloodstream and promoting the formation of atherosclerotic plaques (Liu et al. 2020) activating pathways such as nuclear factor kB signaling (Liu et al. 2020). Oral pathogens as *Campylobacter rectus, Porphyromonas gingivalis, Porphyromonas endodontalis, Prevotella intermedia,* and *Prevotella nigrescens* have been identified in the atherosclerotic lesions, promoting plaque formation. Furthermore, oral pathogens can release toxins with proatherogenic effects such as leukotoxin from *Aggregatibacter actinomycetemcomitans* and gingipains from *Porphyromonas* species (Zhang et al. 2010; Ruan et al. 2023)*.* Patients with cases of periodontitis have elevated levels of LDL and triglycerides and decreased levels of HDL, which are risk factor for STEMI, confirming that oral microbiome have an important role in cardiovascular health (Jaramillo et al. 2013).

Our previous study documented the composition OM in STEMI patients, and bacteria such as *Prevotella* was associated with increased levels of IL-6 and periodontitis (Hernandez-Ruiz et al. 2023). However, the gut microbiota (GM) was not analyzed in that study, and there was no control group to compare the composition. While there have been reports examining the OM and GM in STEMI, most studies have focused on one niche. Therefore, the aim of this study was to associate the composition of the OM and GM with soluble inflammatory mediators and SCFAs in Mexican patients with STEMI.

## 2 Material and methods

### 2.1 Study participants

**Fig. 1:**
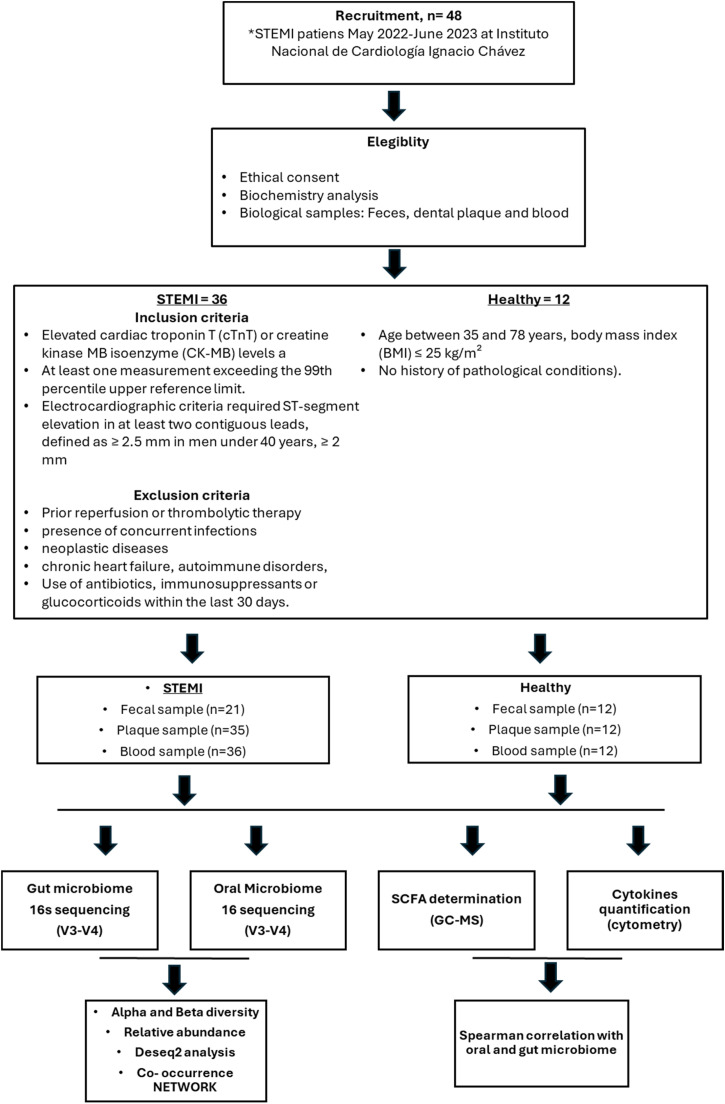
Study flow chart.

This observational study was conducted in the coronary care unit of a specialized cardiovascular clinical center of National Institute of Cardiology “Ignacio Chávez” in Mexico City, between May 2022 and June 2023. **The** Fig. 1 shows the flow chart of study. In detail, we invite to participate adult patients admitted within 24 hours of experiencing a STEMI episode, as defined by the Fourth Universal Definition of Myocardial Infarction (Thygesen et al. 2019). The STEMI diagnosis was confirmed by the presence of anginal symptoms and evidence of myocardial injury, indicated by elevated cardiac troponin T (cTnT) or creatine kinase MB isoenzyme (CK-MB) levels, with at least one measurement exceeding the 99th percentile upper reference limit. Additionally, the electrocardiographic criteria required ST-segment elevation in at least two contiguous leads, defined as ≥ 2.5 mm in men under 40 years, ≥ 2 mm in men aged 40 years and older, or ≥ 1.5 mm in women in leads V2-V3, and/or ≥ 1 mm in other leads, in the absence of left ventricular hypertrophy or left bundle branch block (Ibanez et al. 2018).

Exclusion criteria included prior reperfusion or thrombolytic therapy, the presence of concurrent infections, neoplastic diseases, pregnancy or puerperium, chronic heart failure, autoimmune disorders, or the use of antibiotics, immunosuppressants or glucocorticoids within the last 30 days.

For comparison, we enrolled age matched healthy volunteers without any comorbidities who met the next criteria (age between 35 and 78 years, body mass index (BMI) ≤ 25 kg/m² and no history of pathological conditions). Each participant provided approval to take biological samples as is described next.

In detail, 36 STEMI patients admitted at “Instituto Nacional de Cardiologia Ignacio Chavez” between May 2022- June-2023 that accepted and signed consented inform were included in this study. 21 fecal samples and 35 plaque samples were collected, as well 36 blood samples corresponding to each participant. Oral and gut microbiome were determined using 16s sequencing (V3-V4), SCFAs and cytokines were evaluated by gas chromatography-mass spectrometry and cytometry respectively.

### 2.2 Biological samples

Peripheral blood samples were collected from patients using polyethylene terephthalate tubes (BD Vacutainer) containing polymer gel. The serum was separated by centrifugation and stored at -80°C. Dental plaque (DP) samples were collected by scrapping the vestibular and lingual surfaces of all teeth. The collected samples were placed in Eppendorf tubes containing 70% ethanol and stored at -20°C. Fresh fecal samples were also collected during the patient’s hospital stay and were immediately stored at -80°C.

### 2.3 Circulating short chain fatty acids evaluation by GC-MS analysis

SCFA quantification was performed using an Agilent GC-MS system composed of a 5973 single quadrupole mass spectrometer equipped with an electron ionization (EI) source, a 6890 gas-chromatograph and an autosampler. SCFAs were analyzed in their free acid form using an Agilent J&W DB-FFAP column 30 m length, 0.25 mm internal diameter and 0.25 µm of film thickness by using the oven temperatures’ program, as follows: initial temperature of 50°C helf for 1 min, ramped to 250°C at 30°C/min and maintained for 6.67 min. A 1 µL aliquot of the extracted sample was injected in splitless mode (splitless time 1 min) at 280°C, while the transfer line temperature was 250°C. Helium was used as the carrier gas at a constant flow rate of 1 mL/min. The EI ion source was set to 230°C, with an electron energy of 70 eV, while the focusing lenses were adjusted during the standard tuning routine. The MS acquisition was carried out in single ion monitoring (SIM) by applying a proper dwell time (30 ms for each ion monitored) to ensure an acquisition frequency of at least 4 cycles per second. The quantitative determination of SCFAs in each sample was carried out by the ratio between the area abundance of the analytes and the area abundance of the respective labeled internal standard (isotopic dilution method). The value of this ratio was named Peak Area Ratio (PAR) and it was used as an abundance of each analyte in the quantitative evaluation. The ionic SCFAs’ signals and the reference internal standards used for the quantitation of each SCFAs were reported in the supplementary table 1. Calibration curves were generated for each analyte by plotting PAR, calculated from the ion signals of the analyte and the corresponding internal standard, against its nominal concentration in the calibration solution A linear regression analysis was applied to obtain the best fitting function between the calibration points. In order to obtain a reliable limit of detection (LOD) and limit of quantitation (LOQ) values, the standard deviation of response and slope approach was employed. The estimated standard deviations of responses of each analyte were obtained by the calculated standard deviation of y-intercepts (SDY-I) of regression lines.

### 2.4 Cytokines’ quantification

Serum cytokines were quantified using the LEGENDplexTM Human Essential Immune Response Panel kit, 13plex (Biolegend) that included the following cytokines: IL-2, IL-4, IL-6, IL-10, IL-17A, CXCL-8, CXCL-10, IL-1β, MCP-1, tumor necrosis factor (TNF), interferon-γ (IFN-γ), IL-12p70 and transforming growth factor-β1 (TGF-β1). Samples were evaluated using a FACSAria flow cytometer (Biosciences, San Jose, CA, USA) and raw data were analyzed with the LEGENDplex™ Data Analysis Software Suite (https://legendplex.qognit.com). Each assay was performed by duplicate.

### 2.5 DNA extraction and 16s rRNA sequencing

DNA extraction from DP samples was performed using the EZ-10 Spin Column Genomic DNA Minipreps kit, Animal (Bio Basic Inc). DNA extraction from fecal samples was conducted using the QIAamp PowerFecal Pro DNA Kit (QIAGEN). The DNA concentration, purity and integrity were verified using a spectrophotometer (Nanodrop 1000, ThermoFisher) and gel electrophoresis (ChemiDoc Image System, BioRad). The extracted DNA was sent to Novogene, where libraries where prepared and sequenced using the NovaSeq 6000 PE250 platform (Illumina) with V3–V4 primers: 341F (CCTAYGGGRBGCASCAG) and 806R (GGACTACNNGGGTATCTAAT).

### 2.6 Bioinformatics and Statical Analysis

Demultiplexed raw FastQ files were processed using QIIME2, v.2023.5. The DADA2 plugin was employed to merge, denoise and construct a table of operational taxonomic units (OTUs). Taxonomy assignment was conducted using the SILVA v.132 database at 99 % identity (Quast et al. 2013). Rarefaction was performed at a sampling depth of 50.000 sequences per sample. Shannon and Chao1 indexes were calculated using phyloseq R package (version 1.42.0) and Bray-Curtis dissimilarity was analyzed using Principal Coordinates Analysis (PCoA). Permutational Multivariate Analysis of Variance (PERMANOVA, 999 permutations) was applied, and graphs were constructed with ampvis2 R package (V 2.8.9). Relative abundances graph with the more abundant genus and phylum were constructed with ampvis2 package, as well, a Venn diagram illustrating the overlap between the OM and GM. Differential abundance analysis was performed with DESEQ2, using its default parameters.

Statistical analysis was performed using chi square test for nominal variables and the Kluskal-Wallis test for quantitative variables. The Shapiro-Wilk normality test was performed to all quantitative variables. All analyses were two-tailed, and a significance level of p ≤ 0.05 was considered statistically significant. For correlation analysis, a Spearmen analysis was applied to oral and gut microbiota with SCFAs and cytokines. The heatmap was constructed with only significant association using pheatmap R package (Version: 1.0.12). For the construction of microbiota network, Sparse InversE Covariance estimation for Ecological Association and Statistical Inference (SpiecEasi, R, version 1.1.3) was used. Prefiltering relative abundances (>0.375 %) was performed prior to analysis. Neighborhood selection method (lambda min ratio = 0.01, lambda iterations= 20) was applied to calculate the coefficients regressions and used as edge weights to construct the network using igraph R package (version 2.1.4).

## 3 Results

### 3.1 Clinical data

Demographical and clinical features of STEMI patients and healthy subjects are reported in Table 1. Specifically, we recruited 36 STEMI patients (32 males, 4 females; median age at enrolment of 57± 9.68 years) and 12 subjects (9 males, 3 females; median age at enrolment of 55 ± 11.58 years).

**Table 1:** Demographical and clinical features of enrolled STEMI patients and Healthy subjects. Median (IQR) for quantitative variables, frequency for qualitative variables. U-Mann Whitney test (^a^) and Fisher’s Exact test (^b^) were performed to obtain p-value. P-values less than 0.05 were considered statistically significant.

	**Healthy (n=12)**	**STEMI (n=36)**	p
**Age (years)**	55 (16)	57 (16)	0.93^a^
**Male**	9	33	0.386^b^
**Medical History**			
**Diabetes (total)**	0	15	
**Hypertension (total)**	0	12	
**Active Smokers (total)**	0	17	
**Biochemical analysis**			
**Leucocyte (x103/ul)**	4.5 (1.45)	14.37 (4.27)	0.000*^a^
**Albumin (g/dl)**	4.61 (0)	4.16 (1)	0.000^*a^
**C reactive protein (mg/l)**	0.506 (1.62)	3.61 (6.3)	0.001*^a^
**Troponins (ng/ml)**	6.29 (5.17)	332 (2438)	0.000*^a^
**Creatine Kinase-MB (ng/ml)**	1.68 (1)	20.50 (34)	0.000*^a^
**Lipid profile**			
**Total Cholesterol (mg/ml)**	193 (34)	174 (75)	0.137^a^
**High Density Lipoprotein (mg/ml)**	48.9 (18)	34.5 (11)	0.007*^a^
**Low Density Lipoprotein (mg/ml)**	125.4 (26)	114.77 (64)	0.226^a^

The prevalence of diabetes and hypertension in the STEMI cohort was 42% and 30%, respectively. Additionally, 48% of the STEMI patients were active smokers.

Laboratory results showed elevated leukocyte counts, C-reactive protein (CRP), troponin, and creatine kinase MB isoenzyme (CK-MB) levels in the STEMI group, while albumin levels were lower. HDL cholesterol was significantly lower in the STEMI patients compared to healthy group.

In terms of oral health, 15 STEMI patients (41.6%) had gingivitis and 18 (50%) had periodontitis. In the healthy group, 6 subjects (50%) were diagnosed with gingivitis, but no cases of periodontitis were observed.

### 3.2 Oral and Gut microbiome composition analysis

**Fig. 2:**
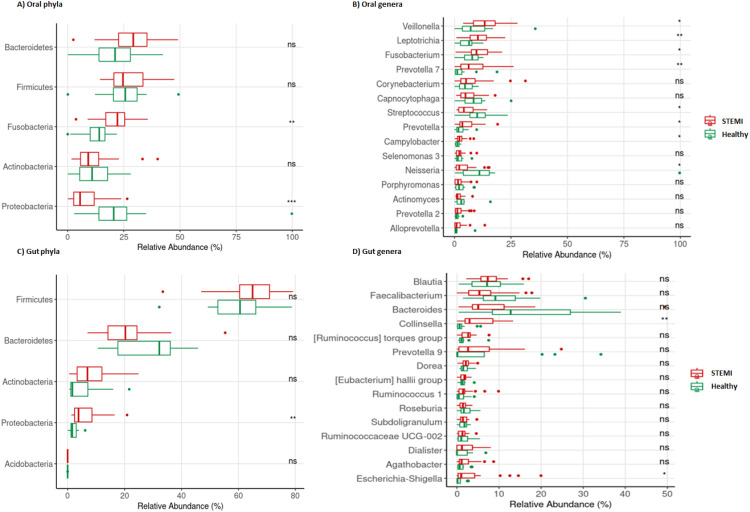
Comparison of relative abundance between STEMI and Healthy groups at the genus and phylum levels. In (A), the five most abundant phyla in the OM are shown, and in (C), the five most abundant phyla in the GM are displayed. In (B) and (D), the 15 most abundant genera are shown in OM and GM, respectively. The Wilcoxon rank-sum test was applied, * (p < 0.05), ** (p < 0.01), *** (p < 0.001).

The composition of the OM and GM at the phylum and genus levels is presented in Supplementary Figures 1 and 2, respectively. When comparing relative abundances between the STEMI and healthy groups, the phylum *Fusobacteria* was elevated in STEMI patients, while *Proteobacteria* was more abundant in healthy subjects (Fig. 2**A**). At the genus level, *Veillonella, Leptotrichia, Fusobacterium, Prevotella 7, Prevotella*, and *Campylobacter* were increased in STEMI patients, whereas *Streptococcus* and *Neisseria* were more prevalent in healthy group (Fig. 2**B**).

For the GM, the phylum *Proteobacteri*a was enriched in STEMI patients (Fig. 2**C**). At the genus level, *Collinsella* spp. were more abundant in healthy group, while *Escherichia-Shigella* spp. showed a higher prevalence in STEMI patients (Fig. 2**D**).

**Fig. 3:**
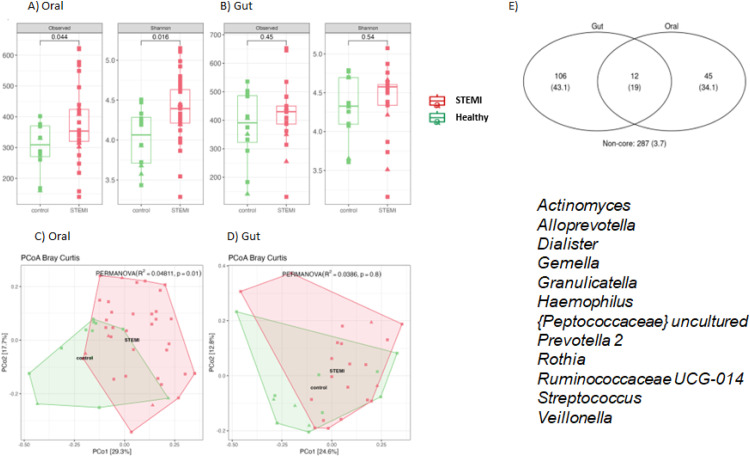
The observed OTUs and Shannon indices were calculated for the OM (A) and GM (B) in both the STEMI and Healthy groups (Wilcoxon rank-test was applied). Principal Coordinate Analysis (PCoA) using Bray-Curtis Dissimilarity and a PERMANOVA test were applied to both the OM (C) and GM (D). A Venn diagram (E) was constructed using the ampvis2 package to identify the core OTUs shared between the Oral and Gut microbiomes. The genera *Actinomyces, Alloprevotella, Dialister, Gemella, Granulicatella, Haemophilus, [Peptococcaceae] uncultured, Prevotella 2, Rothia, Ruminococcaceae UCG-014, Streptococcus*, and *Veillonella* were found in both environments.

Regarding the alpha diversity analysis, STEMI patients showed significantly higher alpha diversity compared to healthy subjects in the OM (observed OTU richness p= 0.044; Shannon index, p=0.016) (Fig. 3**A**). However, no significant differences were observed in GM alpha diversity (Fig. 3**B**). For beta diversity, PCoA using the Bray-Curtis dissimilarity index with a PERMANOVA test revealed significant difference between STEMI and healthy groups in the OM (p = 0.01) (Fig. 3). However, no significant differences were found in the GM (p = 0.8) (Fig. 3**D**).

To explore the overlap between the OM and GM, a Venn diagram based on relative abundance was created to identify core OTUs shared between the two niches. Twelve core OTUs were common to both niches, including *Actinomyces, Alloprevotella, Dialister, Gemella, Granulicatella, Haemophilus, Peptococcaceae* (uncultured), *Prevotella 2, Rothia, Ruminococcaceae UCG-014, Streptococcus*, and *Veillonella* (Fig. 3**E**).

**Fig. 4:**
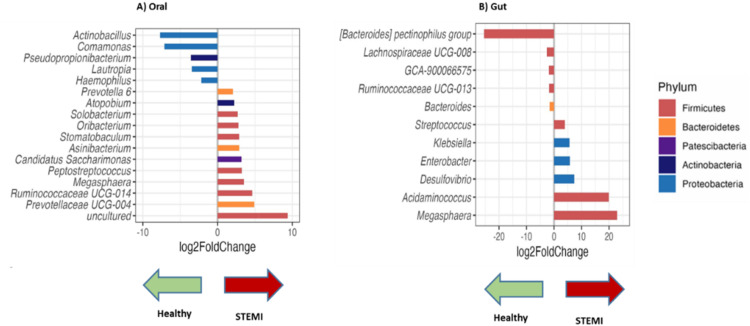
Differential abundance analysis of Oral (A) and Gut (B) Microbiome with DESEQ2 analysis. In both figures, negative fold change is associated with healthy subjects, while positive fold change is associated with STEMI patients.

Differential abundant analysis (DESeq2) was performed for both OM and GM to identify genera that were differentially abundant between the STEMI and healthy groups. In the OM, *Prevotella* 6, *Atopobium, Solobacterium, Oribacterium, Stomatobaculum, Asinibacterium, Candidatus Saccharimonas, Peptostreptococcus, Megasphaera, Ruminococcaceae UCG-014*, and *Prevotellaceae UCG-04* were more prevalent in the STEMI group while *Actinobacillus, Comamonas, Pseudopropionibacterium, Lautropia*, and *Haemophilus* were associated with the healthy group (Fig. 4**A**).

In the GM, the genera *Streptococcus, Klebsiella, Enterobacter, Desulfovibrio, Acidaminococcus*, and *Megasphaera* were more prevalent in the STEMI group. Meanwhile, the genera *[Bacteroides] pectinophilus* group, *Lachnospiraceae UCG-008, GCA-90066575, Ruminococcaceae UCG-013*, and *Bacteroides* were found to be associated with the healthy group (Fig. 4**B**).

### 3.3 Circulating short chain fatty acid profile and correlation with oral and gut microbiome

Table 2 presents the SCFA values for both STEMI and Healthy groups. The last showed a significantly increased abundance of isovaleric acid, while levels of isobutyric and 2-methylbutyric acids were significantly lower compared to STEMI patients.

**Table 2:** Circulating SCFAs levels of Healthy and STEMI patients. Comparisons were assessed with the Wilcoxon rank test and p-values less than 0.05 were considered statistically significant.

**Abundance of each circulating SCFA** (mean (IQR); µmol/L)	**STEMI**	**Healthy**	**p-value**
Total SCFAs	67.20 (38.41)	114.98 (64.08)	0,8418
Acetic acid	49.60 (33.75)	90.89 (50.95)	0,8546
Propionic acid	8.24 (5.03)	7.94 (1.72)	0,3687
Butyric acid	2.65 (1.43)	2.85 (0.00)	0,0751
isoButyric acid	3.35 (1.81)	10.13 (4.81)	0,0001
isoValeric acid	1.41 (0.28)	1.06 (0.55)	0,0187
Valeric acid	0.88 (0.49)	0.87 (0.48)	0,1142

**Fig. 5:**
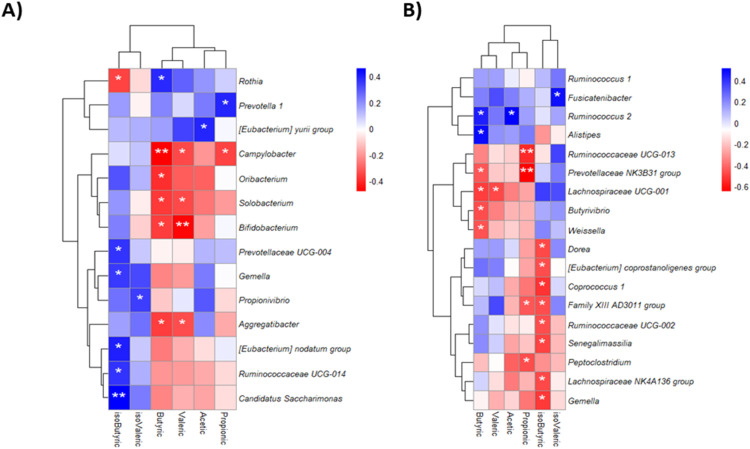
Spearman correlation of SCFAs with OM (A) and (B) GM in STEMI patients. *(p<0.05), ** (p<0.01), *** (p<0.001).

A Spearman correlation analysis was performed to explore associations between SCFA levels and the composition of the OM and GM. In OM, acetic acid was positively correlated with *Eubacterium yurii* spp., while propionic acid was positively associated with *Prevotella 1* spp. and negatively with *Campylobacter* spp. Butyric acid showed positive correlations with *Rothia* spp. and negative correlations with *Campylobacter, Aggregatibacter, Oribacterium, Solobacterium*, and *Bifidobacterium genera*. Additionally, valeric acid was negatively correlated with *Campylobacter, Aggregatibacter, Solobacterium*, and *Bifidobacterium* genera; while isobutyric acid showed positive correlations with *Ruminococcaceae UCG-014, Gemella, Candidatus Saccharimonas, [Eubacterium] nodatum* group, and *Prevotellaceae UCG-004* genera, and negatively with *Rothia* spp. Lastly, Isovaleric acid was positively correlated with *Propionivibrio* spp. (Fig. 5**A**).

For the GM, acetic acid was positively correlated with *Ruminoccocus* while propionic acid was negatively associated with *Peptoclostridium, Ruminococcaceae UCG-013, Family XIII AD3011 group* and *Prevotellaceae NK3B31 group.* Moreover*,* butyric acid showed a positive correlation with *Ruminococcus* 2 and *Alistipes*, but a negative correlation with *Butyrivibrio, Lachnospiraceae, Weisella* and *Prevotellaceae NK3B31 group* genera*.* Finally, valeric acid negatively correlated with *Lachnospiraceae UCG-001* spp. and isovaleric acid positively correlated with *Fusocatenibacter* spp. (Fig. 5**B**).

### 3.4 Cytokines’ profile and correlation with OM and GM genera

**Fig. 6:**
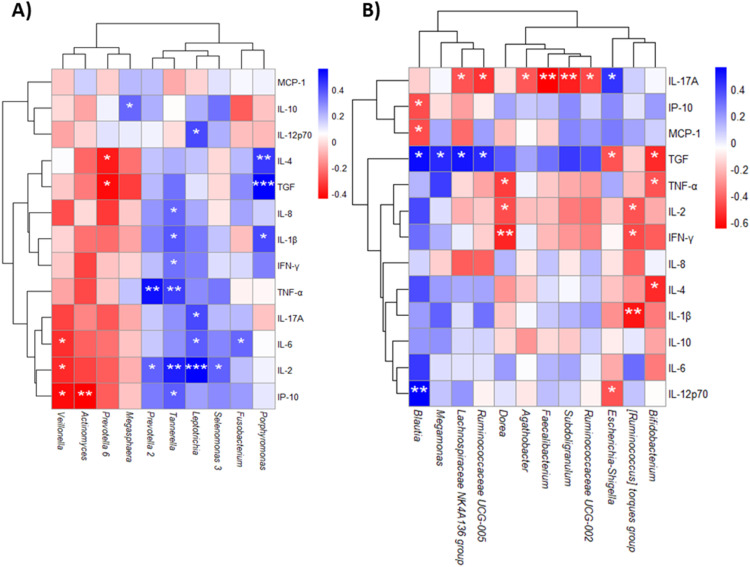
Spearman correlation of cytokines with Oral (A) and (B) Gut microbiome of STEMI group. Spearman correlation was calculated between cytokines and the more relevant genus in both oral and gut microbiome. *(p<0.05), ** (p<0.01), *** (p<0.001).

Serum cytokines were measured in STEMI patients, and the mean values of each cytokine presented in Supplementary Table 1. Spearman correlation analysis was performed to investigate the relationships between serum cytokines and key genera from both the OM and GM. In the OM, TNF-α showed a positive correlation with *Prevotella 2* and *Tannerella*, while IL-1β was positively correlated with *Tannerella* and *Porphyromonas*, both of which are well-known periodontal pathogens. *Veillonella*, a genus linked to periodontal disease, showed a negative correlation with IP-10, IP-2, and IL-6. *Leptotrichia*, a genus associated with caries, positively correlated with IL-2, IL-17A, IL-6, and IL-12p70, while *Porphyromonas* also positively correlated with both IL-4 and IL-1β (Fig. 6**A**).

In the GM, *Blautia* spp. exhibited a negative correlation with IP-10 and MCP1, while *Faecalibacterium*, an SCFA-producing genus, negatively correlated with IL-17A. Other SCFA producers, such as *Dorea* spp. showed a negative correlation with IL-2 and TNF-α, while *Bifidobacterium* spp. negatively correlated with IL-4 and IL-2. Additional SCFA-producing genera, including *Agathobacter, Subdoligranulum, Ruminococcaceae UCG-002, Lachnospiraceae NK4A136* and *Ruminococcaceae UCG-005*, were negatively correlated with IL-17A. Lastly, *[Ruminococcus] torques group*, associated with gut eubiosis, displayed a negative correlation with IL-2 and IL-1β. Conversely, *Escherichia-Shigella*, a bacterium associated with gut dysbiosis and elevated in STEMI patients, displayed a positive correlation with IL-17A (Fig. 6**B**).

### 3.5 Oral and Gut microbiome network

To investigate the connection between OM and GM, a network analysis was performed using the Sparse Inverse Covariance Estimation for Ecological Association and Statistical Inference (SpiecEasi) package.

**Fig. 7:**
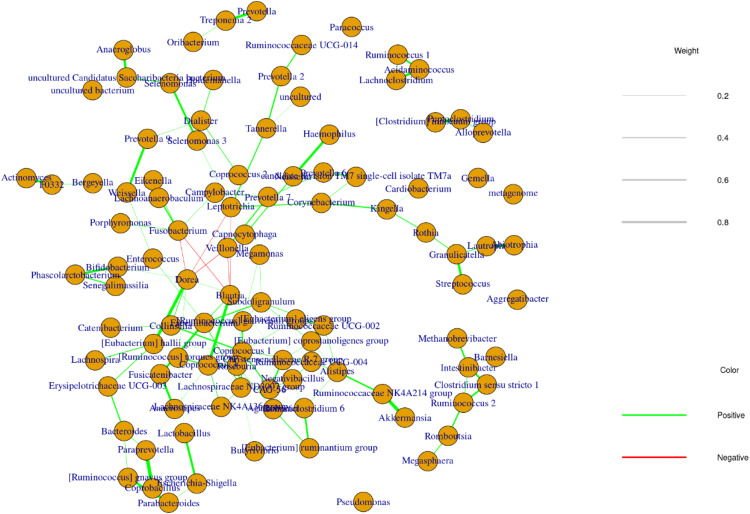
Network among OM and GM of STEMI and Healthy groups was constructed using the SPIEC-EASI (SParse InversE Covariance Estimation for Ecological Association Inference) software. Green lines represent positive association, while red lines represent negative associations. Network was constructed with OTUs with a prevalence > 0.04. The Analysis was run by the neighborhood selection (MB) algorithm with a lambda min ratio of 0.01 and a lambda path of 20 iterations per permutation.

This analysis revealed intricate interactions between various bacteria, as shown in Fig. 7. A prominent cluster was identified, consisting of several SCFA-producing bacteria such as *[Ruminococcus] gnabus group, [Eubacterium] halii group, Ruminococcus 1, Lachnospira, Roseburia, Dorea, Faecalibacterium, Lachnospiraceae, CAG-56, Fusocatenibacter, Faecalibacterium, Agathobacter, Coprococcus* and *Christensenellaceae R-7 group.* The presence of this cluster suggests that these bacteria likely play a crucial role in maintaining gut homeostasis through the production of SCFAs.

Of note, *Clostridium sensu stricto 1* and *Intestinibacter*, both pathogenic genera, were found to interact with the SCFA-producing *Ruminococcus 2* genus. Similarly, *Escherichia-Shigella*, a known pathogenic genus, interacts with *Lactobacillus* spp., which serves as a connection point to the SCFA-producing bacterial cluster. These interactions suggest that *Clostridium* spp. and *Escherichia-Shigella* spp. might disrupt the balance and function of SCFA-producing bacteria in the gut. In addition, periodontal disease-related genera like *Prevotella, Veillonella, Fusobacterium, Leptotrichia, Capnocytophaga* and *Tannerella* were found to form their own interconnected network. Notably, this group also interacts with the SCFA-producing cluster, suggesting a potential influence of poor oral health on the composition and function of gut SCFA-producing bacteria.

## 4 Discussion

In our study we found some GM and OM bacteria associated with STEMI patients. By comparing relative abundance between STEMI and healthy groups, we documented, at the phylum level, that *Proteobacteria* was enriched in STEMI patients, which is consistent with previous reports linking this phylum to cardiovascular diseases (Han et al. 2021; Sawicka-Smiarowska et al. 2021). While, at the genus level, *Escherichia-Shigella* spp. was enriched in STEMI patients, while *Collinsella* spp. was more abundant in healthy individuals. *Escherichia-Shigella* has been associated with cardiovascular disease (Kwun et al. 2020; Clark et al. 2010; Zhu et al. 2018; Luedde et al. 2017), and although *Collinsella* is often reduced in heart failure (Luedde et al. 2017), it is linked to several conditions such as type 2 diabetes, atherosclerosis, arthritis, and inflammatory bowel disease (Lupu et al. 2023) (Wang et al. 2022; Masoodi et al. 2020).

One of the metabolites that support the atherosclerosis development is the Trimethylamine-N oxide (TMAO). Its precursor, trimethylamine (TMA), is produced by disbalanced gut microbiota, and therefore, converted to TMAO in the liver by the FMO3 oxidase. Entering in the bloodstream TMAO can favor a systemic inflammation through some specific pathways such NF-κB. TMAO levels increase in GM dysbiosis, characterized by decreasing SCFA-producers, and increasing of bacteria as *Escherichia-Shigella*, which was found increased in STEMI patients. We documented that several SCFA-producing bacteria were enriched in the healthy group, notably *Ruminococcaceae UCG-013* and *Bacteroides.* The genus *Ruminococcaceae UCG-013* has been reported to promote obesity resistance in mice (Feng et al. 2022) and has been found to be elevated in subjects without hypertension (Maruyama et al. 2023). The *Bacteroides* genus, especially species like *Bacteroides fragilis*, produces polysaccharide-A, a potent immunomodulator with beneficial effects on gut health (Zafar and Saier 2021). Studies have shown that *Bacteroides* spp., such as *Bacteroides acidifaciens*, can increase in response to diets rich in fiber and acetate, providing protection against hypertension and heart failure (Marques et al. 2017). However, *B. fragilis* has been associated with metabolic dysfunction and atherosclerosis reducing the beneficial *Lactobacillus* spp in the gut, and increasing pathogenic bacteria like *Desulfovibrio spp*. (Shi et al. 2022). These divergent roles of *Bacteroides* members suggest that the effects are species-dependent and influenced by complex factors. In our study, the presence of *Bacteroides* spp.in the healthy group supports its protective role in this Mexican cohort, however further analysis needs to confirm the *Bacteroides* species that we detected.

Another key GM metabolite is indole -3 propionate, that can affect the GM composition by increasing α-diversity; additionally, this metabolite can revert the GM dysbiosis, protecting against oxidative damage and reducing inflammation. The main producers of indole propionic acid are species of *Clostridium* genus, like C*lostridium sporogenes, Clostridium botulinum*, and *Clostridium caloritolerans, and s*ome species of *Peptostreptococcus* such as *P. asaccharolyticus* (Jiang, Chen, and Gao 2022). We did not observe changes in these GM species, but at more specific screening is needed to better define the role of these bacteria in Mexican STEMI patients.

On the other hand, the STEMI group showed enrichment of genera linked to cardiovascular disease, such as *Acidaminococcus, Deselfuvibrio* and *Megasphaera*. In line with our findings, these genera were also found to be elevated in Chinese patients with AMI (Han et al. 2021). Additionally, both this study and the one on Chinese AMI patients observed reduced abundance of SCFAs-producing bacteria from the *Lachnospiraceae* family, such as *Lachnospiraceae UCG-008* and *ND3007* (Abdugheni et al. 2022), reinforcing the idea that these taxa have a protective role against myocardial infarction.

Furthermore, we found increased levels of *Streptococcus, Klebsiella,* and *Enterobacter* genera in STEMI patients*;* all of which have been implicated in cardiovascular diseases. For instance*, Enterobacter hormaechei* has been identified in atherosclerotic tissue (Rafferty et al. 2011), and both *Streptococcus* species and *Klebsiella pneumoniae* have been detected in atherosclerotic lesions in patients with coronary heart disease (Ott et al. 2006). It is likely that these bacteria translocate from the gut to the bloodstream, and subsequent accumulation in atherosclerotic plaques, contributing to disease progression (Mao et al. 2024).

Regarding the oral microbiome, our previous study (Hernandez-Ruiz et al. 2023) characterized the OM and cytokine profiles in STEMI patients, documenting that those with worse oral health, particularly periodontitis, exhibited higher alpha diversity in their OM compared to those with gingivitis. In this study, we found that alpha diversity was significantly higher in STEMI patients than in healthy individuals, suggesting that oral dysbiosis in STEMI may be linked to a broader microbial ecosystem in DP. However, it is important to note that many studies have reported decreased ecological OM diversity in systemic diseases associated with dysbiosis (Pisano 2023). This discrepancy might be attributed to the choice of sample site (Roca et al. 2024), which plays a crucial role in determining OM composition. In both our current and previous study, DP was the sample source, differing from other studies that collect samples from saliva or soft tissues for DNA sequencing. DP forms a biofilm on the solid tooth surface, creating microenvironment that fosters a complex microbial community with unique interactions and growth conditions (Valm 2019). Aerobic bacteria in the plaque can consume oxygen, allowing pathogenic anaerobic bacteria, linked to systemic diseases like cardiovascular conditions, to thrive in this oxygen-depleted environment (Mark Welch et al. 2016). Oral bacteria such as *Streptococcus* spp.*, Porphyromonas* spp.*, Tanerrella* spp.*, Prevotella* spp. and *Campylobacter* spp.; which are implicated in both periodontal disease and cardiovascular conditions (Chhibber-Goel et al. 2016), flourish under anaerobic conditions, potentially explaining the observed increase in microbial diversity in patients with worse oral health.

When analyzing individual taxa, we observed an oral enrichment of bacteria belonging to Fusobacteria and Proteobacteria phyla in STEMI and healthy subjects, respectively. The Fusobacteria phylum includes strict anaerobes, with *Fusobacterium* and *Leptotrichia* as its dominant genera (Dewhirst et al. 2010). These bacteria are well-known for their association with various periodontal diseases, including caries and gingivitis (Thurnheer et al. 2019; Kalpana et al. 2020). *Fusobacterium* spp., in particular, have also been linked to systemic conditions such as cancer, atherosclerosis, inflammatory bowel disease and AD (Fan et al. 2023), highlighting their broader impact on health beyond the oral cavity. Conversely, *Proteobacteria* were more abundant in the OM of healthy group. While some reports suggest that *Proteobacteria* are elevated in individuals without caries (Nomura, Otsuka, et al. 2020; Jiang et al. 2018), others associate this phylum with oral diseases, such as oral squamous cell carcinoma (Radaic et al. 2023). This apparent dual role of *Proteobacteria* reflects the phylum’s diversity, encompassing a wide array of both commensal and pathogenic bacteria (Rizzatti et al. 2017). At the genus level, STEMI patients showed significant enrichment of *Veillonnella, Leptotrichia, Fusobacterium* and *Prevotella*, all of which are linked to oral diseases (Le et al. 2023; Liu et al. 2020; Jonsson et al. 2020; Nordendahl et al. 2018).

By using DESeq2, we identified several genera enriched in the OM of healthy and STEMI patients. Among healthy individuals, *Actinobacillus, Comamonas, Pseudopropionibacterium, Lautropia*, and *Haemophilus* genera were enriched. *Lautropia* spp., known to thrive on the DP of healthy subjects, is often less abundant in patients with gingivitis or periodontitis (Huang et al. 2021). Similarly, *Haemophilus* spp. has been observed in greater abundance in healthy individuals compared to those with oral cancers (Unlu et al. 2024), and *Actinobacillus* spp. shows similar patterns (Pushalkar et al. 2011). *Comamonas* spp., while linked to oral dysbiosis in various diseases (Calheiros Cruz et al. 2022; Jiang et al. 2018), has an unclear role as an oral pathogen. On the other hand, *Prevotella 6* spp. and *Atopobium* spp. wer*e* enriched in the STEMI group. These genera have been associated with various diseases, such as gastrointestinal disorders and hypertension (Mohammed et al. 2024; Kononen et al. 2022), suggesting their involvement in systemic disease. Additionally, we found increased abundance of several SCFA-producing genera in the STEMI group, including *Ruminococcaceae UCG-014* and *Oribacterium* (Chin et al. 2022; Liu et al. 2021), along, with caries associated genera, *Stomatobaculum, Candidatus Saccharimonas, Peptostreptococcus* and *Megasphaera* (Radaic and Kapila 2021; Lee et al. 2021; Rams et al. 1992; Kumar et al. 2005; Kumar et al. 2003).

To understand the role of other confounding variables like the presence of hypertension, obesity and active smoking, we performed a comparison of relative abundances in the STEMI patients grouped by these variables (**Supplementary Figure 3**), where Escherichia-shigella only found associated with STEMI patients with hypertension. This confirmed the role of *Escherichia spp.* in gut dysbiosis and in STEMI development.

The translocation of oral bacteria to the gut and systemic circulation has been implicated in cardiovascular diseases. For instance, in caries-induced mice, *Streptococcus mutans* was found in injured heart tissue, with a correlation to its abundance in the oral cavity (Nomura, Matayoshi, et al. 2020). *Veillonella* spp. has been identified in atherosclerotic plaques (Koren et al. 2011), while *Prevotella* species were detected in coronary thrombi of STEMI patients (Sardu et al. 2021). These findings suggest that oral disease-associated bacteria play a critical role in the pathogenesis of STEMI by translocating to systemic sites.

In addition, we identified oral and gut bacteria associated with SCFA and cytokine levels. In the OM, *Tannerella* and *Porphyromonas* species positively correlated with IL-1β, a cytokine known to be elevated during heart damage in STEMI patients. IL-1β activates the pro-inflammatory NF-κB pathway, leading to the production of additional pro-inflammatory cytokines and acute-phase proteins, which are implicated in atherosclerosis and AMI. Additionally, *Tannerella* and *Prevotella* species positively correlate with TNF-α, another cytokine elevated in STEMI that contributes to the production of IL-6 and IL-1 (Mahmoud et al. 2019). Previously, our group reported correlations of oral *Veillonella* spp. and *Leptotrichia* spp. with inflammatory mediators associated with IP-10, IL-2, IL-17A, and IL-6, all of which contribute to the pathogenesis of STEMI. All these genera are associated with periodontal diseases (Jiang et al. 2018). Regarding the GM, we primarily observed negative associations between pro-inflammatory cytokines (IL-17A and IL-1β) and SCFA-producing bacteria such as *Faecalibacterium* spp., *Bifidobacterium spp.* and *Ruminococcus* spp. (Fusco et al. 2023). The only positive correlation identified was between *Escherichia-Shigella* spp. and IL-17A, a known pro-inflammatory cytokine. Species of *Escherichia-Shigella* have molecular mechanisms that can alter GM composition and inhibit the growth of beneficial bacteria, contributing to dysbiosis and conditions such as leaky gut (Allsopp et al. 2020).

SCFAs may play contrasting roles depending on their localization. In the oral cavity, elevated SCFA levels have been linked to tissue damage, which could explain the harmful effects observed in cases of poor oral health, as several oral pathogens produce SCFAs, including *Porphyromonas gingivalis, Fusobacterium nucleatum, Aggregatibacter actinomycetemcomitans, Veillonella parvula*, and *Streptococcus sanguis* (Leonov et al. 2023). The presence of *Megasphaera, Ruminococcaceae*, and *Oribacterium* genera in the oral cavity in STEMI patients suggests these bacteria may contribute to the inflammatory state associated with STEMI. Likewise, *Stomatobaculum spp*., *Peptostreptococcus spp*., and *Candidatus Saccharimonas* spp. likely produce SCFAs due to their association with genes related to fermentative metabolism, characteristic of SCFA-producing bacteria (Biddle 2013).

In addition, we documented that *Eubacterium yurii group* and *Prevotella 1* correlated with acetic and propionic acids, respectively. Species of *Eubacterium* genera are linked to periodontal diseases, while some species *Prevotella* can contribute to oral inflammatory processes. These results suggest that SCFAs in the oral cavity can contribute to local inflammatory processes (Leonov et al. 2023). This can be explained since SCFAs affect negative effects on periodontal cells, inducing pyroptosis and apoptosis mechanism (Magrin et al. 2020). Acetate, propionate and butyrate exhibit toxicity to human gingival cells (Pollanen and Salonen 2000). In addition, SCFAs can alter the expression of several molecules linked to epithelial barriers, such as, Keratin 17, ICAM-1, connexin 23 & 46, cadherin-1 and claudin 1 & 4 (Leonov et al. 2023), leading to increased epithelial permeability.

The relationship between isobutyric-acid and health has not been well studied. This Branch-chain fatty acid is produced in lower quantities by microbiome; however, their function has not been established. We observed that *Ruminococcaceae UCG-014, Eubacterium nodatum* group, *Prevotellaceae UCG-004*, and *Gemella* species positively correlated with isobutyric acid. *Ruminococcaceae UCG-014* spp. was associated with the STEMI group. While the species of *Ruminococcaceae* family are recognized as SCFA producers. *Ruminococcaceae UCG-014* has been linked to diseases like autism inflammatory bowel disease and breast cancer (Zhang et al. 2022; Evenepoel et al. 2024).

In contrast, SCFAs has beneficial effects on the gut, by reducing systemic inflammation and decreasing risk for CAD, mainly via the GPR41 receptor pathway (Tazoe et al. 2008). Acetate, propionate and butyrate are the main SCFAs produced by the gut microbiota, and the decrease of such bacteria, has been linked to the development of systemic diseases like atherosclerosis.

We found a positive correlation between butyrate levels with the genera *Ruminococcus 2* and *Alistipes* which are considered butyrate producers (Peterson et al. 2022). Contrary, *Prevotellaceae NK3B31, Lachnopiraceae UCG-001, Butyryvibrio* and *Weisella* anti-correlate with butyrate. These taxa are considered beneficial for the gut due are considered butyrate producers and is expected the butyrate production in the gut.

However, SCFAs were measured in serum samples; SCFAs are produced in the gut, and a part of their amounts are removed by the feces. So, is needed to quantify both serum and fecal SCFA levels to better understand the role of these bacteria in the butyrate levels.

Finally, several genera were negatively correlated with Isobutyric acid. These genera correspond to *Dorea, Eubacterium coprostanoligenes, Coprococcus 1* and *Ruminococcaceae UCG-002,* that are well known SCFAs’ producers and have health benefits (Fusco et al. 2023). The fact that isobutyric acid positively correlates with oral pathogens and negatively with gut beneficial bacteria indicates that it has a potential negative role on STEMI pathophysiology. Anyways, the role of isobutyric acid is not finely clarified; in detail, it has been linked to diseases like cancer and diabetes, but in meantime it is shown that it enhance the anti-tumoral effect for PD-1 treatment and confers resistance to Inflammatory bowel disease (Dabek-Drobny et al. 2022; Murayama et al. 2024). We recorded increased levels of isobutyric acid on the healthy group; however further studies are requested to better identify the right role of isobutyric acid in human health.

Another key component in the SCFAs metabolism is succinate. This metabolite is a process intermediate of indigestible carbohydrates into SCFAs, which provides an energy source for epithelial cells. Bacteria like *Bacteroides spp. Prevotella spp, Veilonella pp.* and *Firmicutes spp.* can produce succinate starting from pentose and hexose (Wei et al. 2023). In the oral cavity, we documented genera, like *Veilonella* and *Prevotella* that can produce succinate and that are found elevated in STEMI patients. On the other hand, the phylum *Bacteroidetes* along the genus *Bacteroides* are found higher in the healthy group. Similar to other metabolites, succinate has a controversial role. It has been used as target in probiotic interventions to treat obesity and related comorbidities (Li et al. 2023). In fact, succinate is elevated in metabolic and inflammatory diseases, including ischemic heart disease, hypertension, type 2 diabetes and obesity (Fernandez-Veledo and Vendrell 2019). In addition, it is reported that succinate can cause hypertrophy in cardiomyocytes through GPR91 receptor (Yang et al. 2016). In addition, Caucasian Spanish obese showed elevated levels of succinate, and it was associated with a higher relative abundance of *Prevotellaceae* and *Veillonellaceae* (Serena et al. 2018).

Another metabolite related to SCFAs metabolism is methylmalonic acid (MMA). This compound is a byproduct of propionate metabolism, due to vitamin B12 deficiency. Under normal conditions, MMA is present at low levels in the host; however, genetic deficiencies in enzymes of the propionate pathway or GM alterations can lead to elevated MMA levels (Tejero, Lazure, and Gomes 2024). The MMA accumulation has been linked to various pathological effects, including neurodegeneration and kidney failure. In STEMI patients, increased MMA levels have been detected in biological samples such as urine and are considered a marker of elevated myocardial infarction risk (Dhar et al. 2023). The gut microbiota likely plays a key role in regulating MMA levels. Dietary interventions aimed at reducing propionate amounts and promoting the production of other SCFAs, such as butyrate, may help lower MMA levels reducing cardiovascular risk.

To explore the interaction between the OM and GM, we conducted a Venn analysis to identify taxa common to both ecological niches. The most important genera identified in both microbiomes were *Veillonella, Streptococcus* and *Prevotella,* which are associated with periodontal diseases (Dame-Teixeira et al. 2021; Abram et al. 2022; Kononen et al. 2022)*.* In STEMI patients we observed an enrichment in the relative abundance of both oral *Veillonella* and *Prevotella* species, which may translocate to the gut, potentially contributing to alterations in GM composition. Similarly, *Streptococcus spp., a common* oral genus, was also enriched in the GM of STEMI patients, suggesting possible oral-gut translocation.

Our network analysis revealed interactions between *Prevotella* and *Veillonella* species in STEMI patients. These taxa negatively interacted with a cluster of SCFA-producing bacteria, which have anti-inflammatory and health-promoting effects. This suggests that the enrichment of oral disease-associated bacteria may disrupt gut microbial homeostasis and impair SCFA production, contributing to inflammation and disease progression. Furthermore, *Streptococcus* spp. interacted with *Granulicatella, Rothia* and *Lautropia* species. *Rothia* spp. was present in both oral and gut niches and is typically a commensal organism in the oral cavity. However, certain strains can act as opportunistic pathogens, occasionally causing infections like endocarditis (Haddad et al. 2021). On the other hand, *Lautropia* spp., primarily associated with oral health, colonizes the oral cavity and upper respiratory tract and was enriched in healthy subjects in our study (Colombo et al. 2009). Moreover, a cluster of interactions among *Lautropia* spp. and *Streptococcus, Granulicatella*, and *Rothia* species was found in the healthy group. The enrichment of both *Lautropia* and *Streptococcus* species in the healthy group suggests that this cluster may play a significant role in maintaining oral health. While several *Streptococcus* species are linked to dental caries (Nomura, Matayoshi, et al. 2020), others, such as *Streptococcus salivarius* and *Streptococcus thermophilus*, are utilized as probiotics to enhance oral health by reducing the abundance of oral pathogens.

This network analysis suggests that oral bacteria, especially those linked to periodontal disease, can potentially affect GM by interacting with SCFA-producing bacteria. These findings highlight the intricate connection between oral and gut health, where disruptions in OM may lead to alterations in gut microbial communities, potentially impacting overall gut health and SCFA production. This interconnected relationship underscores the importance of maintaining good oral health to support a balanced and functional GM.

## 5 Conclusion

Overall, this study represents the first report examining the oral and gut microbiome in Mexican patients affected by STEMI, contributing valuable insights into the characterization of both oral and intestinal microbial communities. In detail, we identified key bacteria that modulate the microbiome along the oral-gut axis, such as *Veillonella* spp. and *Prevotella* spp. Furthermore, we observed distinct bacterial profiles associated with STEMI patients versus healthy individuals. For instance, oral bacteria like *Lautropia* and *Streptococcus* species were enriched in healthy subjects, while several caries-associated bacteria were enriched in STEMI patients. Our findings highlight the potential for developing preventive strategies against infarction through interventions targeting the oral-gut microbiome. By understanding the intricate relationships between these microbiomes, we may be able to identify novel therapeutic approaches to reduce cardiovascular risk and improve overall health outcomes.

Surely, our study shows some limitations, including the low taxonomic resolution of the V3–V4 region of the 16S rRNA gene sequencing (which limits the identification of specific bacterial species), as well as a small sample size that may obscure potential associations between the oral and gut microbiomes. Additionally, confounding factors such as diet and systemic comorbidities, such as diabetes and hypertension, may affect the results, since the healthy group does not include patients with comorbidities. A study with a larger sample size is needed to elucidate the role of other comorbidities in the composition of the oral and gut microbiome in STEMI patients. But, despite these limitations, we identified, for the first time in STEMI patients, key bacterial taxa in both the oral and gut microbiomes, as well as its association with SCFAs and cytokines. Therefore, further research is essential to finely elucidate these distinct microbial interactions and their implications for cardiovascular disease prevention and management in STEMI patients.

## Ethical approval statement

The study protocol was approved by the local ethics committee (Comité de Ética del Instituto Nacional de Cardiología Ignacio Chávez; protocol number 18-1089) and adhered to the Declaration of Helsinki. Informed consent was obtained from patients or their legal representatives, permitting the use of blood samples and clinical data for research purposes.

## Author Contributions

**LACJ** conducted the study, analyzed the data and wrote the manuscript; **ORAV, PHR, LACJ** and **AREM** collected the samples and perform the cytokine quantification; **PHR** and **LACJ** extracted the DNA samples; **PHR** perform the 16s data analysis; **MP, GB** and **SB** performed gas chromatography-mass spectrometry experiments; **HGP** and **LAG** supervised and contributed to recruitment, diagnosis and analysis of clinical parameters; **LAG, AA** and **MAG** conceived and supervised the protocol design, review and edit the manuscript. All authors contributed to the article and approved the submitted version.

## Declaration of competing interest

The authors declare that they have no known competing financial interests or personal relationships that could have appeared to influence the work reported in this paper.

## Data Availability

The sequences used in this study can be found in: https://www.ncbi.nlm.nih.gov/sra/PRJNA1259703.

## Appendix Supplementary materials

Image, application 1
