# Supplementary material for: The link between inflammatory/ SCFA profiles and oral/gut microbiome: an observational study in patients with ST-segment elevation myocardial infarction
Source: Curr Res Microb Sci. 2025 Jun 15;9:100423. doi: 10.1016/j.crmicr.2025.100423 (PMC12221840; doi:10.1016/j.crmicr.2025.100423)
Supplement: Supplementary file 1 [file mmc1.docx]

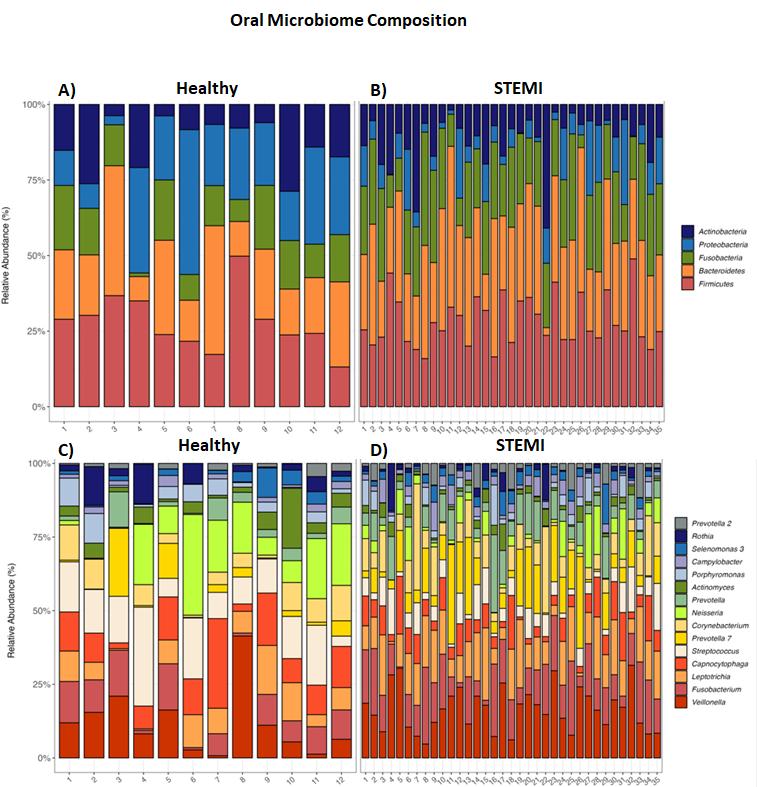


**Supplementary Figure 1**. Composition of the oral microbiome. Relative abundance of phyla (A) and genus(B) in the STEMI and Healthy groups.


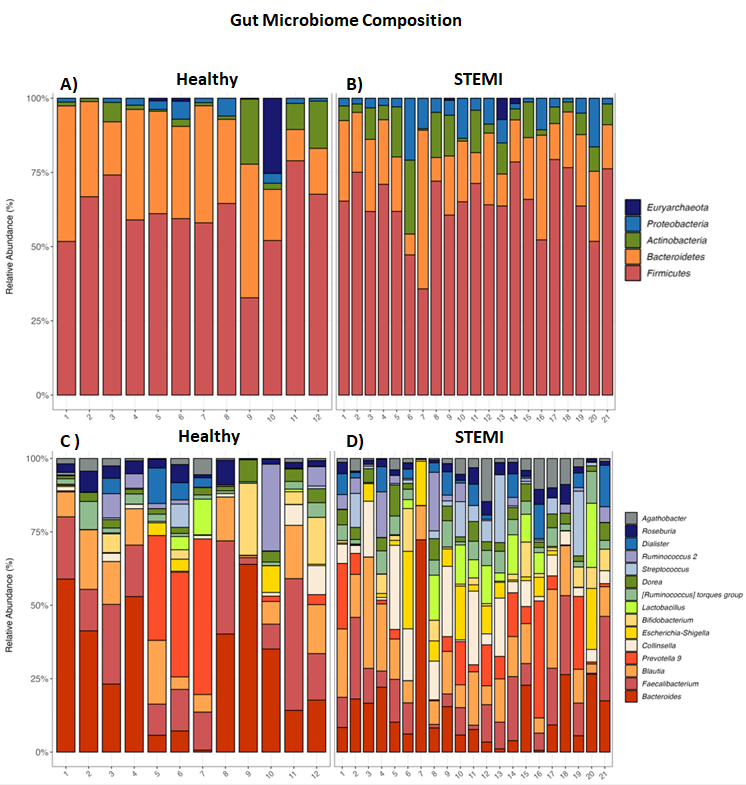


**Supplementary Figure 2.** Composition of the gut microbiome. Relative abundance of phyla (A) and genus(B) in the STEMI and Healthy groups.
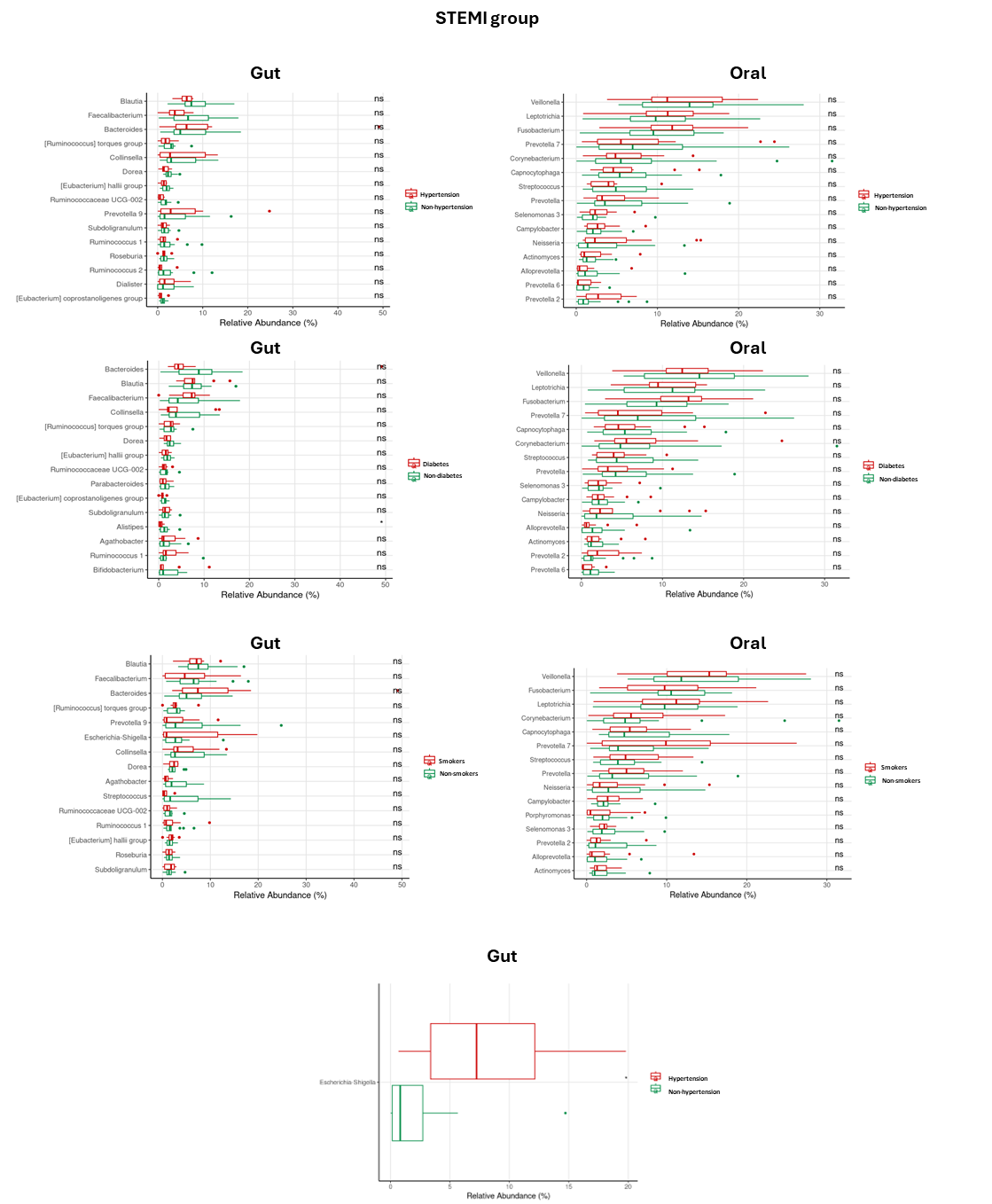


**Supplementary Figure 3.** Relative abundance of oral and gut microbiota in STEMI patients, grouped by hypertension, diabetes, and smoking status. *Escherichia-Shigella* is found to be elevated in STEMI patients with hypertension.

| **Cytokine** | **pg/ml** |
| --- | --- |
| IL-4 | 98.22 ± 198.5 |
| IL-2 | 36.57 ± 54.76 |
| IL-1 | 13.71 ± 17.05 |
| TNF-α | 32.68 ± 62.16 |
| IL-17A | 59.75 ± 64.40 |
| IL-6 | 400.42 ± 269. |
| IL-10 | 54.26 ± 85.89 |
| IFN-γ | 103.33 ± 132.7 |
| IL-12p70 | 66.81 ± 62.62 |
| IL-8 | 24.42 ± 18.21 |
| TGF-β | 552.24 ± 486.1 |
| MCP-1 | 385.51 ± 132.8 |
| IP-10 | 291.78 ± 208.5 |

**Supplementary table 1**. Quantification of cytokines in the STEMI Group.
